# Intelligence in Education

**DOI:** 10.3390/jintelligence7010008

**Published:** 2019-03-04

**Authors:** Tomoe Kanaya

**Affiliations:** Department of Psychology, Claremont McKenna College, Claremont, CA 91711, USA; tkanaya@cmc.edu

**Keywords:** education, IQ, multiple intelligence, SAT, ACT

## Abstract

The articles in this special issue provide an overview of the wide breadth of questions and methodologies that arise when the research devoted to intelligence intersect with the research devoted to U.S. education. The unique contributions of each paper are highlighted and discussed based on their contribution to the literature. The implications of these findings for educational research and policy are briefly discussed.

The use of standardized tests within education in the United States is historically fraught with controversy. At a national level, assessments such as the National Assessment of Educational Progress and Trends in International Mathematics and Science Study are used to determine the academic achievement of public school children within and between schools and states over time. There is more than mere “bragging rights” at stake. For example, accountability standards included in specific educational policies can mean that schools are denied federal funds based on their students’ performance on these assessments [1]. 

At the individual level, a student’s performance on intelligence measures is used in many decisions that affect his or her day-to-day experience in the classroom. Much of this is due to the Individuals with Disabilities Act (IDEA; initially the Education for All Handicapped Children Act), which is more commonly referred to as special education [2]. Under IDEA, all students who meet the criteria for the 13 categories in the Code for Federal Regulations (CFR) [3] are entitled to ‘free and appropriate’ educational services. Each diagnostic criteria, however, requires an IQ test. Furthermore, every student who receives services is required to undergo a re-evaluation at least every three years, thereby making school children in special education one of the most heavily tested and re-tested populations in the United States on IQ tests.

The articles by Beaujean, Benson, McGill, and Dombrowski [4] and Kanaya and Ceci [5] illustrate the complexity in how IQ is used and interpreted within IDEA. Beaujean et al. [4] provide insights on the use of the IQ-achievement discrepancy model for the diagnosis of Learning Disability, the most prevalent disorder served under IDEA [6]. Kanaya and Ceci [5] examine the impact of the Flynn effect on the initial evaluation and required re-evaluation of students diagnosed with Emotional Disturbance. While Emotional Disturbance is less prevalent than Learning Disability, students who are served under this diagnosis usually require more costly services [6], and therefore, can have a substantial impact on schools. From these papers, it is clear that IDEA is an important aspect of education that requires future examination from intelligence researchers. For example, conducting cross-sectional and longitudinal work on students who remain in IDEA compared to students who test out before graduation as well as examining the relationship between IQ and other achievement tests on all of the CFR diagnoses will also provide valuable insights for researchers, policy-makers, practitioners, and administrators. 

Shearer’s [7] paper reviews neurological data that support and expand upon Gardner’s theory of multiple intelligences. In addition to summarizing key findings, Shearer goes on to reshape Gardner’s theory into a paradigm that can aid in teacher preparation and classroom instruction, and combines cognition, socio-emotional development, cultural context, and neuroscience. This approach promotes a multi-dimensional perspective to student achievement including individual differences in intelligence(s), and is particularly promising in combating the criticisms of group differences including racial and gender differences [8] on many intelligence measures used in schools such as IQ and standardized achievement tests. Age-based norms on all of the intelligences, however, are needed for educators to monitor and compare student development and growth over time and should be a focus for future research in this area. 

Finally, the paper by Wai, Brown, and Chabris [9] focuses on the relationship between intelligence, higher education, and labor market outcomes. By using the SAT and ACT scores at an institutional level, their results focused on the outcomes that went beyond compulsory, K–12 education. Given the push for public schools to prepare their students to be “college and career ready” [10] and the majority of high school graduates enroll in college [11], it is clear that students in higher education are an important population to include in education research. Unlike primary and secondary schools where enrollment eligibility is determined by geographical location, colleges and universities are able to select their students based on individual qualities and characteristics (e.g., age, race, gender, religious affiliation). This selection process allows for a wide range of research on the relationship between environmental and genetics on individual differences in adulthood outcomes. 

Readers of this journal will be familiar with the myriad of ways in which intelligence is defined and the controversies that surround these definitions. Indeed, these papers illustrate: (1) the same definition of intelligence (e.g., IQ) can be used differently in a manner that directly affects the day-to-day educational experiences of individuals, especially children with disabilities; (2) an individual’s intelligence and intelligences must be considered when creating optimal learning environments; and (3) intelligence plays an important role in the relationship between an individual’s education and post-educational outcomes. These papers, however, also point to a need to develop an appropriate definition, or set of potential definitions, for “education”. More specifically, when does education start and when does it end? Has the purpose of an education changed in the United States and what data are required to determine if that purpose is/has been met? How do the goals of education change based on individual characteristics versus group characteristics versus environmental characteristics? These are just a few of the questions that can help future researchers create a more comprehensive understanding of the role of intelligence in education. 
